# Calcium Signaling in Brain Cancers: Roles and Therapeutic Targeting

**DOI:** 10.3390/cancers11020145

**Published:** 2019-01-26

**Authors:** Ahmed Maklad, Anjana Sharma, Iman Azimi

**Affiliations:** Division of Pharmacy, College of Health and Medicine, University of Tasmania, Hobart, Tasmania 7001, Australia; aaettm@utas.edu.au (A.M.); asharma2@utas.edu.au (A.S.)

**Keywords:** calcium signaling, brain cancers, therapeutic targeting

## Abstract

Calcium signaling, in addition to its numerous physiological roles, is also implicated in several pathological conditions including cancer. An increasing body of evidence suggest critical roles of calcium signaling in the promotion of different aspects of cancer, including cell proliferation, therapy resistance and metastatic-related processes. In many cases, this is associated with altered expression and/or activity of some calcium channels and pumps. Brain cancers have also been the subject of many of these studies. In addition to diverse roles of calcium signals in normal brain function, a number of proteins involved in calcium transport are implicated to have specific roles in some brain cancers including gliomas, medulloblastoma, neuroblastoma and meningioma. This review discusses research that has been conducted so far to understand diverse roles of Ca^2+^-transporting proteins in the progression of brain cancers, as well as any attempts to target these proteins towards a therapeutic approach for the control of brain cancers. Finally, some knowledge gaps in the field that may need to be further considered are also discussed.

## 1. Introduction

Calcium (Ca^2+^) is a secondary messenger involved in a variety of cellular processes, including cell growth, apoptosis, differentiation, metabolism, muscle contraction, neuronal plasticity and gene transcription [1,2]. In addition to its diverse physiological roles, Ca^2+^ signaling is also implicated in various pathological conditions, including cancers. Diverse cellular processes critical for cancer progression are Ca^2+^ signal-dependent, including proliferation, angiogenesis, invasion and metastasis [3]. Recently, Ca^2+^ signaling has also been shown to promote growth and tumorigenesis of brain cancers. Although, most of these studies so far have been in glioblastomas, an increasing body of evidence suggest crucial role of Ca^2+^ signaling in other types of brain cancers too. While the calcium signal is ubiquitous, the proteins that control Ca^2+^ transport are not ubiquitously expressed. Indeed, altered expression and/or activity of specific Ca^2+^ channels and pumps have been shown in cancers. These proteins regulate highly specialized and selective processes which contribute to cancer initiation or progression [1,4]. At the same time, these proteins may not have vital roles in surrounding normal cells. Thus, altered expression or activity of these proteins in cancers can be therapeutically exploited to suppress a pathway that is critical for tumorigenesis. Such therapeutic approaches could be of vital importance in some brain cancer treatment where the current therapeutic options are inadequate or show debilitating side effects. This review discusses roles and therapeutic targeting of Ca^2+^ transporting proteins in brain cancers. 

**Figure 1:**
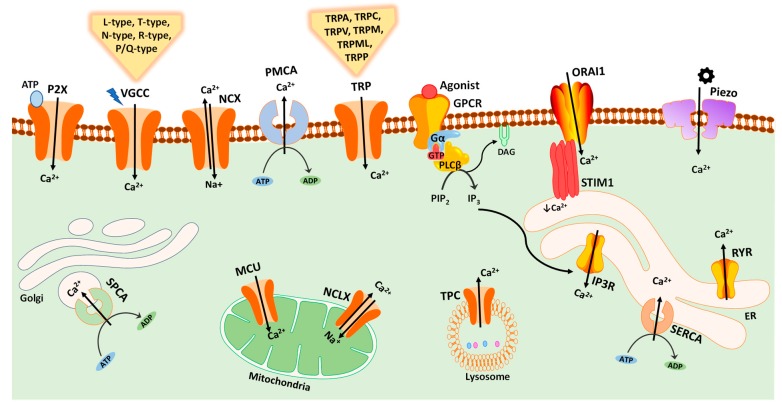
Schematic representation of major calcium channels, pumps, exchangers and sensors in mammalian cells. Ca^2+^ influx is mediated by plasma membrane channels including transient receptor potential (TRP) channels, voltage-gated calcium channels (VGCC), ligand-gated ionotropic P2X receptors, mechanosensitive Piezo channels, and store-operated Ca^2+^ entry pathway mediated by stromal interaction molecule 1 (STIM1) sensor and ORAI1 channels. Distribution of Ca^2+^ against a chemical gradient across cell compartments is regulated by Ca^2+^ pumps including the plasma membrane Ca^2+^-ATPase (PMCA), Sarcoplasmic/endoplasmic reticulum Ca^2+^-ATPase (SERCA), and Golgi network secretory pathway Ca^2+^/Mn^2+^-ATPase (SPCA). The endoplasmic reticulum (ER) Ca^2+^ channels include ryanodine receptor (RYR) and inositol 1,4,5-trisphosphate (IP3) receptor (IP3R); the latter is activated by IP3 ligand produced by the plasma membrane G protein-coupled receptor (GPCR) via Gaq and phospholipase C-β (PLCβ) proteins. Two-pore channels (TPC) regulate Ca^2+^ release from the endolysosomal system. Mitochondrial Ca^2+^ levels are controlled by mitochondrial calcium uniporter (MCU) complex, and mitochondrial Na^+^/Ca^2+^ exchanger (NCLX).

Intracellular Ca^2+^ signals generated by cells are specific in magnitude, time course and location and involve various components of calcium signaling toolkit including channels, pumps, exchangers, signaling proteins and its dependent effectors. Figure 1 shows an overview of the key components of Ca^2+^-transporting proteins, mediating Ca^2+^ transport in or out of cell, and into or out of intracellular organelles. Changes in Ca^2+^ signaling can induce modifications in cell physiology. Therefore, a firm control on Ca^2+^ levels within cells is essential [5]. 

Normal cell functioning requires maintenance of varied distribution of Ca^2+^ against a chemical gradient across cell compartments which is carried out and regulated by energy driven Ca^2+^ pumps. These pumps change the affinity and availability of Ca^2+^ binding sites within cell compartments with variation in Ca^2+^ concentrations [6]. Sarcoplasmic/endoplasmic reticulum Ca^2+^-ATPase (SERCA), plasma membrane Ca^2+^-ATPase (PMCA) and Golgi network secretory pathway Ca^2+^/Mn^2+^-ATPase (SPCA) are the main pumps found in mammals. An upward or downregulated expression of Ca^2+^ pumps is observed in various cancers and is believed to promote differentiation and apoptosis resistance in tumor cells [6]. Ca^2+^ signals are mediated by inositol 1,4,5-trisphosphate (IP3) receptor and ryanodine receptors (RyRs) in sarco and endoplasmic reticulum. Voltage-gated Ca^2+^ channels (VGCC), transient receptor potential (TRP) ion channels, store-operated Ca^2+^ entry (SOCE) channels, and ligand-gated Ca^2+^ channels are involved in movement of Ca^2+^ ion across plasma membrane. For instance, TRP channels, also known as cellular sensors [7], consists of a number of cation channels that mediate cellular functions such as cell growth, apoptosis and motility [8]. Ligand-gated ionotropic P2X receptors are a class of purine receptors that regulate passage of Ca^2+^ into the cell in response to extracellular ATP [9]. Piezo channels are plasma membrane mechanosensitive channels that are stimulated by mechanical forces (Figure 1). A detailed description of components and complexity of Ca^2+^ signaling has been reviewed extensively elsewhere [2,10,11]. Intracellular Ca^2+^ can be derived from both internal (such as endoplasmic or sarcoplasmic reticulum) and external sources (such as via plasma membrane ion channels). Ca^2+^ produces signals in the form of waves and spikes. Spikes activate localized cellular processes, while Ca^2+^ waves occur when individual channels communicate with each other to produce global signals leading to activation of both intercellular (such as functioning of glial cells) and intracellular (such as gene transcription and cell proliferation) functions [12]. Ca^2+^ signals for longer periods are relayed via repetitive Ca^2+^ pulses known as oscillations. Both Ca^2+^ spikes and waves can produce oscillating signals triggering different processes depending upon their signal duration [12].

## 2. Calcium Signaling in Cancer

In recent years, studies elucidating the molecular mechanism involved in cancer have led to an enhanced understanding of various factors that play a role in disease progression and complexity [13]. In an attempt to simplify the complex biology underlying the disease, Hanahan and Weinberg in their review, “The hallmarks of cancer: the next generation”, introduced ten key characteristics of the neoplastic cells. These are maintenance of long term growth signaling, resistance to cell death, angiogenesis induction, metastasis instigation, growth suppressor evasion, uncontrolled proliferation potential, unstable genome and mutation, energy metabolism reprogramming, evasion of immune system and presence of tumor promoting inflammation [14]. Cancer pathology reveals genetic changes in the growth signaling that impair tightly regulated normal cell homeostasis leading to transformation of normal cells into malignant type with uncontrolled proliferative potential [15]. A growing body of evidence suggests that ion channels play a pivotal role in tumor biology and are involved in regulation of tumor cell characteristics such as uncontrolled growth, sustained angiogenesis, metastasis and resistance to apoptosis [16]. In some cases, this regulation occurs via the transmission of signals from the tumor microenvironment. For instance, the involvement of Ca^2+^ channels in regulation of epithelial to mesenchymal transition (EMT) [17], a process critical for cancer metastasis [18,19] and also contributes to drug resistance [20]. 

In tumor cells, altered Ca^2+^ movement often involves aberrant expressions, cellular localization and activity of Ca^2+^-transporting proteins contributing towards tumor specific characteristics such as unlimited growth, metastasis and resistance to apoptosis [3,4]. Since Ca^2+^ signals are vital for normal cell function, alteration in these signals may lead to modified calcium codes that can trigger tumorigenic behavior. Ca^2+^ pulses serve as “calcium codes” differing in magnitude, time and frequency. Prolonged Ca^2+^ pulses are involved in cell division process, angiogenesis, differentiation and genomic instability in cancer cells [21]. 

### Brain Cancers

Brain cancers are neoplasms that originate within the brain and spinal cord mainly from neurons or glial cells (astrocytes, oligodendrocytes and ependymal cells), and occasionally from pituitary gland, meninges, lymphatic tissue and cranial nerves [22]. These tumors exhibit with varying degrees of malignancy and are associated with high mortality and morbidity in both children and adults [22,23]. 

Medulloblastomas (MBs), ependymomas and high-grade gliomas are the most prevalent tumors affecting children and adults [23]. Medulloblastoma, arising from immature neuronal precursors of cerebellum, affects about 25% of pediatric brain cancer patients [24]. For treatment, patients are divided into standard and high-risk groups. High-risk patients have residual tumor mass of >1.5 cm^2^ showing metastasis, whereas low risk patients have residual tumor with the size of < 1.5 cm^2^ without metastasis. Current therapies for medulloblastomas include surgery, radiotherapy and chemotherapy. The use of chemotherapeutic agents as an adjuvant has led to improved results and radiation dose reduction [25].

Ependymomas, accounting for approximately 10% of pediatric brain tumors, originate from ependymal cells that form the lining of the spinal cord and ventricles of the brain [25]. Recent studies indicate that ependymomas have varied genetic aberrations and transcriptional profiles depending upon their location within the central nervous system. Surgery and irradiation remain the mainstay of treatment, as chemotherapy shows limited efficacy in most patients [25,26].

In adults, the most common type of brain tumor is high-grade gliomas. It includes glioblastoma and anaplastic astrocytoma [27]. Current treatment options include maximal surgical resectioning, followed by radiation and adjuvant temozolomide chemotherapy. For anaplastic gliomas, the standard treatment is surgery and radiotherapy, as the efficacy of chemotherapy remains uncertain [27]. 

Despite improving the survival rate of patients, current therapies pose serious complications that impact quality of life in patients. Traumatic injuries such as damage to blood vessels and ischemia are common problems associated with surgical resectioning, while radiation and chemotherapy cause neurocognitive deficits, cerebral necrosis, peripheral neuropathy, myelopathy and secondary neoplasms [28]. Current neoplastic treatments not only cause neurological complications, but also act as precursor to problems such as tumor relapse [29]. Lack of specific targeting of tumor cells by standard care therapies results in damage to normal cells leading to systemic toxicity. Furthermore, their inability to kill cancer stem cells (CSCs), that possess self-renewal properties, leads to appearance of secondary tumors [30]. CSCs are rare population of tumor cells that have the ability to multiply and differentiate via both symmetrical and asymmetrical cell division. These cells play a key role in initiation, growth, maintenance, metastasis and survival of cancers. It is, therefore, essential to develop treatments that specifically target and eliminate CSCs with minimal damage to normal cells for more effective clinical outcomes [31]. Recent studies have indicated the involvement of calcium channels in controlling a number of functions important in CSCs such as cell volume, progression, metastasis and angiogenesis and therefore, deregulated calcium signaling could serve as a target for cancer therapy [32].

## 3. Calcium Signaling in the Brain

Ca^2+^ channels play crucial role in nervous system development. Short-term rise in Ca^2+^ levels, known as calcium transients, are believed to play an important role in mediating various neuronal development stages such as growth, migration, differentiation, survival and formation of neuronal network by maintaining different influx patterns [33]. 

In brain, key functions of neurons are regulated by flow of Ca^2+^ between the intracellular compartments and across plasma membrane in brain. The movement of Ca^2+^ is triggered in response to stimulus such as membrane depolarization, intracellular Ca^2+^ store depletion, mechanical stretch, extracellular agonists and intracellular messengers [34]. 

The influx of Ca^2+^ leads to induction of a number of Ca^2+^ dependent processes such as neurotransmitter release, neuronal excitability, synaptic plasticity, memory storage and gene expression [35,36,37]. This diversity in neural functions mediated by Ca^2+^ signal is a result of distinctive signals that differ in their scale, temporal and spatial properties [34]. Specific changes in the Ca^2+^ concentration results in distinct function modification in the same neuron types depending upon the short, medium and long duration and distance travelled by the signal [34]. 

Ca^2+^ regulated functions involve a set of ion channels, exchangers and pumps present in plasma membrane, mitochondria, endoplasmic reticulum, golgi apparatus and nucleus, collectively known as Ca^2+^ toolkit. These Ca^2+^-transporting proteins together with G protein-coupled receptors, Ca^2+^ binding proteins, and related transcriptional networks mediate functions important for brain physiology [38].

## 4. Ca^2+^-Transporting Proteins in the Progression of Brain Cancers

The following section summarizes the specific roles of Ca^2+^-transporting proteins in brain cancers. Most of these studies are conducted in glioma cells, with some research focusing on medulloblastoma, neuroblastoma and meningioma cells. Table 1 summarizes studies showcasing specific roles of Ca^2+^-transporting proteins in the progression of brain cancers. It should be noted that in this review, we only discuss studies that show direct association of Ca^2+^ transporters and not the ones based on other ion transporters that are Ca^2+^ dependent. This is, exemplified, by the role of calcium-activated potassium channel KCa3.1 in glioma cell migration, invasion and temozolomide resistance [39]. 

### 4.1. Calcium Signaling in Glioma Cells

#### 4.1.1. Store-Operated Ca^2+^ Entry (SOCE) in Glioma Cells

SOCE is a process of Ca^2+^ influx to refill the endoplasmic reticulum’s (ER) Ca^2+^ after depletion. This process is initiated when the reduced levels of ER Ca^2+^ are sensed by STIM1 leading to its oligomerization and interaction with ORAI1 Ca^2+^ channel on the plasma membrane consequently resulting in the opening of the channel and influx of Ca^2+^ [40]. In primary human glioblastoma cells, higher levels of SOCE and ORAI1 expression have been observed in comparison to non-malignant human primary astrocytes [41]. While small interfering RNA (siRNA)-mediated silencing of ORAI1 (but not STIM1) marginally reduces cell proliferation, silencing of both ORAI1 and STIM1 dramatically reduces invasion of glioblastoma cells [41]. In another study, pharmacological inhibition of SOCE and ORAI1 silencing, led to decrease in cell proliferation and induction of apoptosis in glioblastoma cells [42].

#### 4.1.2. Voltage-Gated Calcium Channels (VGCCs) in Glioma Cells

In neurons, Ca^2+^ influx is regulated by VGCCs. Opening of these channels results in high intracellular Ca^2+^ levels that activate processes such as neurotransmitter release, neuron growth and gene expression [43]. VGCCs are divided into three main categories: high voltage activated channels (including P/Q-type, N-type and L-type), intermediate voltage activated R-type channels, and low voltage activated T-type channels (Ca_V_3 channels) [44]. T-type Ca^2+^ channels control processes such as cell proliferation and differentiation by regulating Ca^2+^ levels at low voltage through creation of resting inward calcium current. Aberrant expression of T-type Ca^2+^ channels is observed in diverse tumor types [45]. In human glioblastoma cells, mibefradil, a T-type Ca^2+^ channel blocker with a weak L-type channel inhibiting activity [46], and siRNA-mediated downregulation of CACNA1G (Ca_V_.3.1) and CACNA1H (Ca_V_3.2) reduces cell proliferation, induces apoptotic cell death, and sensitizes cells to ionizing radiation via AKT/mTORC2 axis [47]. In another study, treatment with mibefradil was shown to enhance the efficacy of subsequently administered temozolomide in human glioblastoma xenograft lines grown in immunodeficient mice [48]. More recently, small hairpin RNA (shRNA)-mediated silencing of Ca_V_3.2 or mibefradil treatment was shown to inhibit the proliferation, survival and stemness features of glioblastoma stem-like cells (GSC) as well as sensitize them to temozolomide chemotherapy [49]. This regulation was shown to be mediated by inhibition of pro-survival AKT/mTOR pathway, promotion of proapoptotic survivin and BAX pathways, and modulation of the expression of number of oncogenes and tumor suppressor genes [49]. Mibefradil, taken orally, suppressed growth of GSC-derived xenografts, increased survival and enhanced temozolomide sensitivity [49]. 

#### 4.1.3. TRP Channels in Glioma Cells

TRP channels are expressed in a number of tissues where they control diverse cellular functions such as cell growth, proliferation, differentiation, migration and apoptosis [50]. TRP channels are activated by both chemical and physical stimuli as well as through changes in cell microenvironment [50]. Number of studies have shown the important roles of TRP channels in glioma progression. In D54MG glioblastoma cells, treatment of cells with SKF96365 (a non-selective TRP-canonical (TRPC) channel blocker) led to inhibition of cell proliferation and cytokinesis [51]. In another study, shRNA mediated suppression of TRPC1 or treatment with SKF96365 in D54MG glioma cells, resulted in inhibition of cell proliferation and incomplete cell division [52]. This study also showed that shRNA suppression of TRPC1 in a flank tumor model, reduced tumor size [52]. Further studies demonstrated that motility of D54MG cells stimulated by epidermal growth factor (EGF), is regulated by TRPC1 channels, in a manner that is dependent on lipid raft proteins [53]. Another TRPC member, TRPC6, was shown to have a crucial role in cell growth, clonogenic ability and G2/M phase cell cycle transition, and its inhibition promoted antiproliferative effects of ionizing radiation [54].

Calcium signaling regulates hypoxia-associated pathways, and several Ca^2+^-transporting proteins are shown to interplay with hypoxia-inducible factor-1 (HIF-1), which is master regulator of hypoxia transcriptional responses [55,56]. In glioma cells, TRPC6 is activated by hypoxia, where it contributes to hypoxic elevation of intracellular Ca^2+^. Inhibition of TRPC6, by shRNA or expression of its dominant-negative mutant (DNC6), abolished hypoxia-induced HIF-1α protein accumulation in U251 human glioblastoma cells via promotion of HIF-1α hydroxylation [57]. Hypoxic activation of TRPC6 was also shown to contribute to cell metabolism via regulation of glucose transporter 1 (GLUT1) expression and glucose uptake [57]. In another study, hypoxic upregulation of TRPC6 expression (via Notch signaling), resulted in activation of nuclear factor of activated T-cells (NFAT), proliferation, invasion and angiogenesis of glioblastoma cells [58].

A number of TRP-melastatin (TRPM) channels have been implicated in glioblastoma progression: reactive oxygen species (ROS)-activated TRPM2 channel induces cell death in A172 human glioblastoma cells [59]; TRPM7 channel promotes proliferation and migration of A172 cells possibly through activation of JAK2/STAT3 and Notch signaling pathways [60]; and TRPM8 channel, via activation of the large-conductance Ca^2+^-activated K^+^ membrane ion channels (BK channels), regulates migration of DBTRG glioblastoma cells [61,62].

TRP-vanilloid (TRPV) channels also have important functions in gliomas. Activation of TRPV1 by capsaicin, leads to Ca^2+^ influx and induction of apoptosis in U373 human glioblastoma astrocytoma cells via p38 mitogen-activated protein kinases (MAPK) activation [63]. Interestingly, neural precursor cells, in an antitumorigenic response, migrate to high-grade astrocytoma cells and activate highly-expressed TRPV1 channels on these cells by releasing endovanilloids. This activation results in cancer cell death via activating transcription factor-3 (ATF3)-dependent ER stress pathway [64]. Cannabidiol-induced TRPV2 channel potentiates chemosensitivity of carmustine, temozolomide and doxorubicin in U87MG and MZC cells [65]. TRPV2 was shown in another study, to negatively regulate glioma cell proliferation and survival, and resistance to extracellular signal-regulated kinase (ERK) dependent Fas-induced apoptotic cell death [66]. Another study in in vitro and in vivo models showed that TRPV2 enhances glioblastoma stem-like cells (GSCs) differentiation toward a more mature glial phenotype, and suppresses their proliferation [67].

#### 4.1.4. IP3 Receptors in Glioma Cells

IP3 receptors (IP3R) convert external stimulus to intracellular Ca^2+^ signals of diverse spatial and temporal patterns such as waves and oscillations [68]. In glioblastoma cells, pharmacological inhibition of IP3R3 by caffeine, reduces cell invasion and migration, and increases survival of subject animals [69].

#### 4.1.5. P2X Receptors in Glioma Cells

Members of the P2X receptors have been shown to play roles in different cancer types [70]. In gliomas, several studies have implicated the role of P2X7 receptors. In rat C6 glioma cells, activation of P2X7 receptors promoted cell migration and expression of pro-inflammatory factors [71]. In another study in C6 cells, P2X7 receptor suppression via use of antagonist or shRNA, promoted epidermal growth factor receptor (EGFR) signaling and growth of cells in in vitro, and tumor growth and angiogenesis in rat-transplanted in vivo models [72]. Intriguingly, P2X7 receptor activation in radiosensitive M059J cells resulted in enhanced cell death [73]. Indeed, P2X7 receptor was later proposed as a predictor gene for glioma patient radiosensitivity and survival probability [74]. In 1321N1 astrocytoma cells, P2X7 receptor was shown to activate ERK1/2 phosphorylation via activation of proline-rich tyrosine kinase 2 (Pyk2), c-Src, phosphatidylinositol 3′-kinase (PI3K), and protein kinase C (PKC) [75].

### 4.2. Calcium Signaling in Medulloblastoma Cells

In medulloblastoma cells, proton-sensing ovarian cancer G protein-coupled receptor 1 (OGR1) promotes the expression of TRPC4 channels in transformed granule cells (DAOY cells). TRPC4 channels in these cells (but not in normal cerebellar granule precursor cells), enhances cell motility in wound healing and transwell migration assays [76].

### 4.3. Calcium Signaling in Neuroblastoma Cells

In mouse N1E-115 neuroblastoma cells, using plasmid overexpression, siRNA-mediated silencing or mibefradil treatment, it was shown that Ca_V_3.1 and Ca_V_3.2 promote cell proliferation [77]. Ca_V_3.2 also stimulates differentiation of NG108-15 cells via an autocrine mechanism of facilitating extracellular secretion of differentiation-promoting factors [78].

Full-length and short isoforms of the TRPM2 channels (TRPM2-L and TRPM2-S respectively) are shown to be upregulated in neuroblastoma tissues compared to adrenal glands [79]. SH-SY5Y neuroblastoma cells stably expressing TRPM2-L showed enhanced protection against cell death induced by oxidative stress via increased forkhead box transcription factor 3a (FOXO3a) and superoxide dismutase 2 (SOD2) levels, while cells expressing TRPM2-S showed enhanced levels of ROS and reduced cell viability [79,80]. TRPM2-S expressing SH-SY5Y xenografts showed reduced HIF-1/2α levels and their target proteins, as well as reduced tumor growth [80]. Pharmacological inhibition of TRPM2-L or expression of TRPM2-S enhanced sensitivity of cells to doxorubicin [80]. 

P2X7 receptors, in addition to glioma cells that was discussed above, plays role in neuroblastoma cells too. In neuroblastoma cells lacking trophic support (serum-deprived), P2X7 receptor expression is enhanced via EGFR and PI3K/AKT pathway, where it facilitates cell proliferation [81].

Retinoic acid-mediated differentiation of neuroblastic (N-type) SH-SY5Y cells was shown to be associated with downregulation of SOCE and expression of STIM1 and ORAI1 proteins [82]. Similarly, in another study, an increase in Ca^2+^ efflux and expression of PMCA2, PMCA3 and PMCA4 was observed in differentiated IMR-32 neuroblastoma cells compared to undifferentiated cells [83]. In both these studies, however, it was not discussed if SOCE or PMCA pumps are involved in the induction of differentiation or these altered activity/expression are consequences of differentiation. 

Treatment of neuroblastoma cells with chemotherapeutic agents, cisplatin and topotecan, increased intracellular Ca^2+^ levels over time. Furthermore, expression of S100A6, IP3R1, IP3R3, RYR1, RYR3 were altered upon treatment with cisplatin and topotecan, and pharmacological modulators of Ca^2+^-transporting proteins in combination with these two agents enhanced cytotoxicity [84]. Further studies are needed to determine the specific roles of these Ca^2+^-transporting proteins in neuroblastoma progression. 

### 4.4. Calcium Signaling in Meningioma Cells

VGCCs are shown to promote meningioma cells. The addition of diltiazem and verapamil (mainly L-type calcium channel blockers) to meningioma chemotherapy drug hydroxyurea (HU), and the antiprogesterone, mifepristone (RU486), enhanced cell growth inhibition through induction of apoptosis and G1 cell-cycle arrest in vitro. This approach also decreased tumor size in meningioma subcutaneous mouse flank tumors through suppression of proliferation and microvascular density [85,86]. 

**Figure 2:**
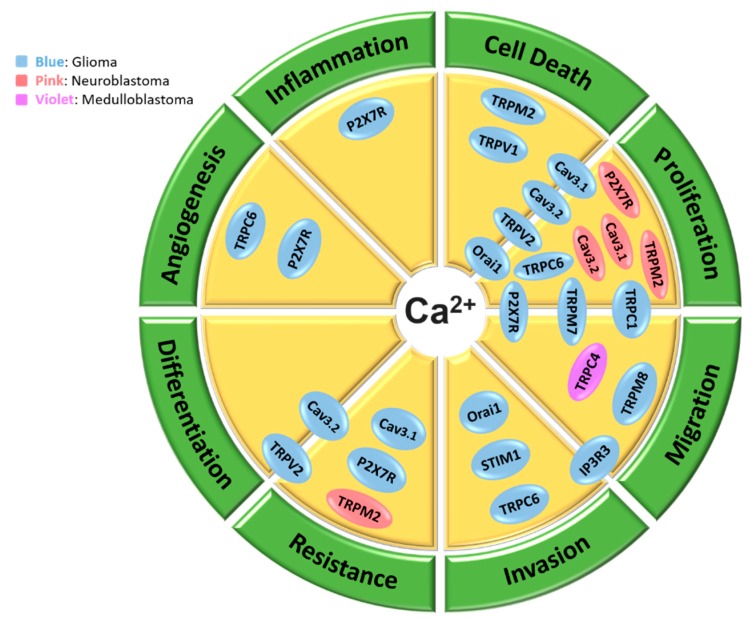
Schematic representation of biological processes that are promoted by Ca^2+^ signaling proteins in brain cancer cells. Several Ca^2+^ channels are shown to contribute to various pro-tumor processes in glioma cells (blue color), neuroblastoma cells (pink color) and medulloblastoma cells (violet color). These processes include proliferation, migration, invasion, therapy resistance/therapy sensitivity, differentiation, angiogenesis, inflammation and cell death. Proteins that are positioned on the borders of two processes, contribute to both processes.

Figure 2 compiles different processes of brain cancer progression assessed in these studies into distinct categories of cellular processes and places each Ca^2+^ channel/regulator into its relevant process where it has shown involvement. These processes include proliferation, migration, invasion, therapy resistance/therapy sensitivity, differentiation, angiogenesis, inflammation and cell death. Many of these processes are involved in cancer hallmarks.

## 5. Targeting Calcium Signaling Pathways as a Therapeutic Approach for Brain Cancers

In the previous section, we discussed studies that have shown important roles of Ca^2+^-transporting proteins in the promotion of different aspects of brain cancer progression. In this section, we discuss studies that are more translational and are conducted in in vivo preclinical models or in clinical trials. 

Table 2 summarizes clinical trial studies that are completed or currently underway in brain cancers using modulators of Ca^2+^-transporting proteins and outlines the outcomes of these studies. Perhaps one of the most important studies, is recent completion of phase II clinical trial of Mipsagargin (also known as G-202) for recurrent or progressive glioblastoma (NCT02067156), as well as for prostate cancer [87]. Mipsagargin which is a SERCA pump inhibitor, is an analogue of thapsigargin. Mipsagargin is a non-toxic prodrug that is activated through binding to and cleavage by prostate-specific membrane antigen (PSMA) that is rich in many cancers [87]. Mibefradil, has been also at the center of attention for clinical trials. Indeed, two clinical trials of phase I were recently completed for assessment of mibefradil in combination with temozolomide (NCT01480050, [88]) or hypofractionated radiation (NCT02202993, [89]) for recurrent glioblastoma. These treatments were well-tolerated in patients and showed some promising responses in selective of patients suggesting that these treatments warrant further investigation [88,89]. A phase I clinical trial was also recently completed for assessment of carboxyamidotriazole orotate (CTO), an inhibitor of non-voltage dependent calcium channels (blocking both the Ca^2+^ influx and release from intracellular stores), for recurrent and newly diagnosed glioblastoma and other anaplastic gliomas (NCT01107522, [90,91]). This study showed safe co-administration of CTO with temoxolomide or chemoradiation, favorable brain penetration and promising signals of activity in patients [90,91]. A Phase II study focusing on combination of verapamil and hydroxyurea (HU) for refractory meningiomas showed no effect of HU or verapamil on tumor recurrence and progression-free survival (PFS) [92]. 

Apart from these clinical studies, there have also been some exciting pre-clinical studies in in vivo models with translational potential. In a recent study, pharmacological targeting of T-type Ca^2+^ channels with niguldipine and mibefradil, induced selective cell death of glioma-initiating cells and enhanced host survival in an orthotopic mouse model of human glioma [93]. In another study, trifluoperazine (TFP), a well-known antipsychotic, inhibited proliferation (via induction of cell death), migration and invasion of glioblastoma cells in vitro, and tumor growth in in vivo xenograft mouse model. This effect was shown to be via direct binding of TFP to the Ca^2+^-binding protein, calmodulin subtype 2 (CaM2), leading to dissociation of CaM2 from IP3R and subsequent extensive and irreversible Ca^2+^ release from endoplasmic reticulum by IP3R1 and IP3R2. Interestingly, this study also showed that TFP has less toxic effects on neural stem cells compared to glioblastoma cells [94]. Bradykinin, a neuropeptide of the vasculature, was shown to induce Ca^2+^ release from internal stores, which ultimately led to increase of glioma invasion. This was shown to be mediated by inducing amoeboid migration via contraction of the cytoskeleton and activation of Ca^2+^-dependent K^+^ and Cl^−^ channels [95]. As also discussed in previous section, another recent study demonstrated novel roles of Ca_V_3.2 in glioblastoma stem-like cells and further supported the use of mibefradil in combination with temozolomide for glioblastoma therapy [49]. Furthermore, in vitro studies in glioblastoma cells demonstrated enhanced radiosensitivity with TRPC6 inhibition [54], while activation of TRPV2 channel increased chemosensitivity of cells [65]. P2X7 receptor activation also led to an increase in radiosensitivity of glioblastoma cells both in vitro [73] and in vivo [74]. 

Altogether, these studies showcase promising approaches of therapeutic targeting Ca^2+^ signaling pathways for the control of brain cancers. It is interesting to note that the therapeutic targeting of plasma membrane Ca^2+^ channels in brain cancers has been focused on the use of blockers of these channels and no study has used channel activators to induce cytosolic Ca^2+^ overload and subsequent cell death, as was recently shown in breast cancer cells through activation of TRPV4 channel [96].

## 6. Conclusions and Perspectives

As discussed throughout the paper, a number of studies suggest involvement of Ca^2+^-transporting proteins in different aspects of brain cancer progression. Interestingly, all the proteins that were identified in these studies were Ca^2+^ channels of different classes (except from STIM1 that is an ER Ca^2+^ sensor). These Ca^2+^ channels included T-type voltage-gated channels, number of TRP channels, ORAI1, IP3R3 and P2X7R. As shown in Figure 2 many of the cellular processes that are regulated by these proteins are involved in cancer hallmarks. It should be noted that these studies identify roles of Ca^2+^ signaling and specific transporting proteins in the *progression* of cancer, however it would be interesting and critical to also assess if calcium signaling and its regulating proteins are involved in the *initiation* of brain cancers. Furthermore, many of these studies are conducted in in vitro models of cancer cells (in most cases continuous cell lines). While these in vitro continuous cell line models provide very important information, these studies need to be also conducted in primary brain cancer cells and in vivo models to have a better understanding of potential translational impact. It is also imperative that in addition to cancer cells, the effect of targeting identified proteins be assessed in normal brain cells, highly sensitive cells that when damaged can result in debilitating permanent side-effects. 

As was shown, majority of the research conducted so far in the field of calcium signaling in brain cancer has been undertaken in glioma cells with most of it in the past few years, underlining that there remain many unanswered research questions in this emerging field. In particular, role of Ca^2+^-transporting proteins in transmitting the signals from the brain tumor microenvironment to cancer cells, is an area of research that requires further investigation. 

Ion channels are reported to represent 18% of all FDA-approved small-molecule drug targets [97]. Given the extensively long and expensive process of drug development, assessment of these existing drugs in brain cancers, alone or in conjunction with current therapies, may provide the opportunity of rapid drug re-purposing for brain cancer therapy. In light of the extensive roles of calcium signaling in normal function of cells, the challenge lies in targeting calcium signaling proteins without causing major side effects. Current advancements in several fields can assist in achievement of this goal, including advancement in development of more selective and less toxic compounds, integrative genomics and transcriptomics studies providing gene maps of brain cancers, and novel drug delivery and activation approaches. These will significantly contribute towards safe and effective therapeutic targeting of Ca^2+^-regulating proteins in coming years.

## Figures and Tables

**Table 1:** Ca^2+^-transporting proteins with demonstrated specific roles in brain cancers.

Cancer	Channel/Regulator	Model	Targeting Approach	Role of Channel/Regulator	References
**Glioma**	**STIM1, ORAI1**	Primary GB cells	siRNA	Regulates proliferation (only ORAI1) and invasion	[41]
	**ORAI1**	U251, C6 cells	siRNA, antagonist	Regulates cell proliferation and apoptosis	[42]
	**Cav3.1, Cav3.2**	U251, U87 and T98G cells	siRNA, antagonist	Regulate apoptosis and proliferation, and sensitize cells to ionizing radiation	[47]
	**Cav3.2**	GB primary stem cells, Xenografted mice	siRNA, antagonist	Promotes proliferation and stemness. Sensitizes cells to TMZ *	[49]
	**TRPC1**	D54MG cells, flank tumor	shRNA, antagonist	Promotes cell proliferation and cytokinesis, as well as tumor size	[52]
		D54MG cells	shRNA, antagonist	Promotes cell motility	[53]
	**TRPC6**	U251, U87, C6 cells, Xenografts	shRNA, DNC6 * antagonist	Promotes cell growth, clonogenicity, and G2/M transition	[54]
		U251 cells	shRNA, DNC6 * antagonist	Induces HIF-1α accumulation and glucose uptake	[57]
		U373, HMEC-1 cells	siRNA	Promotes NFAT activation, cell proliferation and angiogenesis	[58]
	**TRPM2**	A172 cells	Overexpression	Induces cell death	[59]
	**TRPM7**	A172 cells	siRNA	Promotes proliferation and migration	[60]
	**TRPM8**	DBTRG cells	Agonist, antagonist	Promotes cell migration	[61,62]
	**TRPV1**	U373 cells	Agonist	Induces apoptosis	[63]
	**TRPV2**	U87MG, MZC cells	Agonist	Increases chemosensitivity	[65]
		U87MG, MZC cells	siRNA, overexpression	Negatively regulates proliferation and resistance to cell death	[66]
		GB primary stem cells	Antagonist, siRNA, overexpression in xenograft	Promotes differentiation and inhibits proliferation	[67]
	**IP3R3**	U178, U87, T98G cells, Organotypic, Xenograft	Antagonist	Regulates invasion and migration	[69]
	**P2X7R**	C6 cells	Agonist	Promotes migration and inflammation	[71]
		C6 cells, Xenograft	Antagonist, shRNA	Negatively regulates cell proliferation, tumor growth and angiogenesis	[72]
		M059J, GL261 cells	Agonist, antagonist, siRNA	Promotes cell radiosensitivity	[73,74]
		1321N1 cells	Agonist	Promotes ERK1/2 activation	[75]
**MB**	**TRPC4**	DAOY, ONS76 cells, Organotypic	Agonist, antagonist, overexpression	Promotes cell motility	[76]
**Neuroblastoma**	**Cav3.1, Cav3.2**	N1E-115 cells	siRNA, antagonist overexpression	Promotes cell proliferation	[77]
	**Cav3.2**	NG108-15 cells	siRNA	Promotes cell differentiation	[78]
	**TRPM2**	SH-SY5Y cells, Xenograft	Overexpression, antagonist	Regulates cell death/viability	[79,80]
	**P2X7R**	N2a cells	Antagonist	Promotes cell proliferation	[81]
**Meningioma**	**L-type channels**	IOMM-Lee cells, xenograft	Antagonist	Promotes apoptosis and cell-cycle arrest	[85,86]

* DNC6, Expression of the dominant-negative mutant TRPC6; GB, glioblastoma; TMZ, temozolomide; MB, medulloblastoma.

**Table 2:** Modulators of Ca^2+^-regulating proteins in clinical trials for treatment of brain cancers.

Intervention	Channel/Pump Targeted	Disease	Clinical Phase	Study End Year	Results	NCT ^#^	References
Verapamil +Hydroxyurea (HU)	L-type channels	Refractory Meningiomas	II	2015	No effect of HU or verapamil on tumor recurrence and PFS	00706810	[92]
Mipsagargin	SERCA pump	Recurrent or progressive GB	II	2017	Favorable tolerability and pharmacokinetic profile	02067156	[87]
Mibefradil +Temozolomide	T-type channels	Recurrent Glioma	I	2017	Well tolerated and promising responses in patients	01480050	[88]
Mibefradil +Hypofractionated radiation	T-type channels	Recurrent GB	I	2017	Safe co-administration, effective brain penetration, and promising local control signals in some patients	02202993	[89]
CTO +Temozolomide or chemoradiation	Non-voltage channels	GB and other anaplastic gliomas	I	Still active	Safe co-administration, favorable brain penetration, and promising signals of activity	01107522	[90,91]

GB, glioblastoma; PFS, progression-free survival; CTO, Carboxyamidotriazole orotate.
